# Economic Analysis of the Prevention and Control of Nosocomial Infections: Research Protocol

**DOI:** 10.3389/fpubh.2021.531624

**Published:** 2021-07-07

**Authors:** Eric Nguemeleu Tchouaket, Drissa Sia, Sylvain Brousseau, Kelley Kilpatrick, Sandra Boivin, Bruno Dubreuil, Catherine Larouche, Natasha Parisien, Carl-Ardy Dubois, Astrid Brousselle, Roxane Borgès Da Silva

**Affiliations:** ^1^Department of Nursing, Université du Québec en Outaouais, Gatineau, QC, Canada; ^2^Ingram School of Nursing, McGill University, Montreal, QC, Canada; ^3^CISSS des Laurentides, St.Jerome, QC, Canada; ^4^Institut de Cardiologie-Montreal Heart Institute, Université de Montréal, Montreal, QC, Canada; ^5^Department of Nursing, Centre Intégré Universitaire de Santé et de Services Sociaux du Saguenay–Lac-Saint-Jean, Saguenay, QC, Canada; ^6^Department of Biomedical Risks and Occupational Health, Institut National de Santé Publique du Québec, Quebec, QC, Canada; ^7^School of Public Health, University of Montréal, Montreal, QC, Canada; ^8^School of Public Administration, University of Victoria, Victoria, BC, Canada

**Keywords:** economic analyses, costs, case control design, prevention and control, research protocol, prospective observational study, nosocomiai infection

## Abstract

**Background:** Nosocomial infections (NIs) are among the main preventable healthcare adverse events. Like all countries, Canada and its provinces are affected by NIs. In 2004, Ministry of Health and Social Services (MSSS) of Quebec instituted a mandatory surveillance NI program for the prevention and control (NIPC) in the hospitals of the province. One target of the MSSS 2015–2020 action plan is to assess the implementation, costs, effects, and return on investment of NIPC measures. This project goes in the same way and is one of the first major studies in Canada to evaluate the efficiency of the NIPC measures. Three objectives will be pursued: evaluate the cost of implementing clinical best practices (CBPs) for infection control; evaluate the economic burden attributable to NIs; and examine the cost-effectiveness of the NIPC by comparing the costs of CBPs against those of NIs.

**Methods:** This project is based on an infection control intervention framework that includes four CBPs: hand hygiene; hygiene and sanitation; screening; and additional precautions. Four medical and surgical units in two hospitals (nonUniversity, University) in the province of Quebec will be studied. The project has four components. Component 1 will construct and content validate an observation grid for measuring the costs of CBPs. Component 2 will estimate CBP costs *via* 2-week prospective observations of health workers, conducted every 2 months over a 1-year period. Component 3 will evaluate, through a matched case-control study, the economic burden of the four most monitored NIs in Quebec (*C-difficile*, MRSA, VRE, and CPGNB). Archival patient data will be collected retrospectively. Component 4 will determine the optimal breakeven point for CBPs associated with NIPC.

**Discussion:** This project will produce evidence of the economic analysis of NIPC and give health stakeholders an overview of NIPC cost-effectiveness. It will meet the objectives of the Canadian Patient Safety Institute and the MSSS action plan to analyze the efficiency of NIPC preventive measures. To our knowledge, this is the first such exercise in Quebec and Canada. It will provide governments with a decision support tool through a major empirical study that could be replicated nationally to capture the financial benefits of NIPC.

## Background

In recent years, healthcare institutions in Organisation for Economic Co-operation and Development (OECD) countries have been under cultural, regulatory, and, especially, economic pressures. They are confronted with the acceleration of health expenditures (1, 2), problems related to population aging and to medical and technological advances (3, 4), and rising expectations of patients regarding care quality and safety (3). Over this time, standards for governance, leadership, drug management, and infection prevention, and control have been imposed on health facilities (5). The quality and safety of inpatient care is a major issue in health systems. As a result, interest in matters of safety has been growing in recent years (6–11). Health professionals, decision-makers, the public, and scientists are all deeply preoccupied with analyzing patient safety and infection control in healthcare facilities (12).

Nosocomial infections (NIs) are infections acquired during an episode of care in a healthcare facility (13). They are a significant burden on patients, healthcare organizations, and the public health system (14, 15). They lead to increases in care complications, mortality, morbidity, and lengths of hospital stays (16–19). They also generate higher human, material, and financial costs for health facilities (16–18). NIs are one of the main preventable health care adverse events (20). Like every country in the world, Canada is affected by NIs. In 2013, the Public Health Agency of Canada reported that more than 200,000 patients contract nosocomial infections each year, resulting in more than 8,000 deaths. Since 1997, mortality rates due to *Clostridium difficile-associated diarrhea* (CDAD) have tripled in Canada. From 1995 to 2009, the rate of NIs caused by *methicillin-resistant Staphylococcus aureus* (MRSA) bacteremia increased by more than 1,000% (21). In addition, the incidence of *vancomycin-resistant enterococcus* (VRE) in Canada increased significantly from approximately.02 cases per 10,000 patient days in 1999 to approximately.68 cases per 10,000 patient days in 2011 (22).

The province of Quebec is no exception to this reality. To address this persistent problem, the Ministry of Health and Social Services (MSSS) implemented, in response to the 2004 CDAD crisis, standardized mandatory surveillance of NIs in all hospitals in the province. This surveillance is based on a reference program for nosocomial infection prevention and control (NIPC program), which has become the mainstay for healthcare institutions in managing risks, quality, and patient safety. The program is based on seven key actions: (1) surveillance of NIs and monitoring for emergent infectious problems; (2) development of policies, procedures, and support measures; (3) education and training; (4) evaluation; (5) communication and information; (6) outbreak management; and (7) risk management. Its effective implementation should lead to a reduction in NIs (23).

It is with this in mind that decision-makers in the health network wish to evaluate the implementation, costs, effects, and return on investment of the NIPC program. To this end, in target 22 of its 2015–2020 action plan (24) on the prevention and control of NIs for safe delivery of healthcare in the province of Quebec, the MSSS sets out the need to: “evaluate NIPC measures, taking into account the organizational model, the burden of disease, as well as their clinical and epidemiological impacts.” Action 1 of that same target stipulates the need to “assess the clinical and economic impacts of NIs, as well as the outcomes of measures to prevent and control these infections in both general and specialized hospitals” (25). This action by the MSSS is in line with the approach that has been observed since the last economic recession in 2008 in terms of controlling public spending.

The issue of infection prevention investment cost is undeniably very relevant and timely. Moreover, since 2010, Canada has shown great interest in assessing costs associated with adverse events and nosocomial infections (26). In fact, the Canadian Patient Safety Institute funded a project entitled *The Economic Burden of Patient Safety* to undertake research to assess the economic impact of adverse events and the potential benefits for patients and the healthcare system of reducing them (12). Meanwhile, the province of Quebec did not stand idly by. In 2011, the public health directors of the MSSS initiated a study on the economic benefits of public health interventions. This project, led by the Charles LeMoyne Hospital Research Centre under the direction of Professor Astrid Brousselle and Eric Tchouaket, was aimed at developing robust, economically sound arguments to demonstrate the efficiency (economic value) of certain public health programs in Quebec. The programs studied were chosen by public health directors of Quebec at their provincial round table. This is how the project on the economic analysis (EA) of the NIPC program of Quebec came into being. The present research project is part of the same strategy. This is the first major study in Quebec to evaluate the efficiency of an NIPC program. Several benefits are envisaged. On the clinical front, this project will inform healthcare providers and facility managers about the benefits of rigorous application of NIPC program measures, as well as about the costs of managing a patient who is either a carrier of, or infected with, an NI. On the political front, it will provide a strong evidence-based rationale for NIPC investment in Quebec and Canada. It will, thereby, help the MSSS achieve target 22 of its 2015–2020 action plan. On the research front, this project is a precursor to the development of EA studies on both public health and nursing interventions.

## Objectives

The main objective of the project is to evaluate the efficiency of an NIPC program by conducting an economic analysis of clinical best practices (CBPs) implemented. Three specific objectives will be pursued:

1. Evaluate the cost of implementing CBPs associated with NIPC;
2. Assess the economic burden attributable to NIs;
3. Examine the cost-effectiveness of NIPC by comparing the cost of implementing CBPs against the costs of NIs.

## Main Hypothesis

This research project is based on the hypothesis that proper implementation of measures related to clinical best practices for prevention and control of NIs is efficient. In other words, investing in these measures is both beneficial in terms of health and economically profitable.

## Methods

### Theoretical Framework

This project builds on the adverse-events prevention framework developed by Resar et al. (27) at the Institute for Healthcare Improvement in the United States, which advocates implementing “bundles” of three to five evidence-based clinical best practices (CBPs) in professional teams. These practices ensure healthcare providers can, together, provide safe care for their patients. This intervention framework supported the implementation, in Canadian and Quebec healthcare facilities, of infection prevention and control strategies and the deployment of Canadian (28) and Quebec (29, 30) safe care campaigns. According to the Public Health Agency of Canada, the use of best practices for prevention would reduce the risk of contracting some NIs to near zero (21). The CBPs for NIs are subdivided into four essential measures: (1) hand hygiene; (2) hygiene and sanitation of surfaces and equipment; (3) screening on the admission of patients who are carriers or infected, in accordance with the protocols of the healthcare facility; and (4) application of additional precautions. Appendix 1 presents a diagram of the project framework based on the CBPs associated with NIPC.

**i) Hand hygiene**. Hand hygiene refers to the washing and disinfecting (hygienic and surgical) of hands, wrists, and forearms using water, soap, and hydroalcoholic solutions (HAS) at four specific times (see Appendix 2). This action extends from the initial wetting of the hands to the point where they are completely dry (31). The World Health Organization (WHO) estimates that hand hygiene could help reduce healthcare-associated infections by 50%.

**ii) Hygiene and sanitation of surfaces and equipment**. In Québec, a 2005 report from the MSSS committee studying NIPC, entitled *D'abord, ne pas nuire* [*First, Do No Harm*], stressed the importance of cleanliness and sanitation as one of the fundamental measures for infection prevention and control (32). Neglect of regular preventive cleaning and disinfection of surfaces and equipment creates a reservoir for the spread of microorganisms. Hygiene and sanitation activities must be carried out with appropriate frequency (daily, or several times daily), depending on the risk area (33, 34).

**iii) Screening on admission of patients who are carriers or infected**. Screening is the systematic search, in persons with or at risk for NI, for any hitherto undetected condition or anomaly, based on screening algorithms (Appendix 3). Screening techniques differ according to the type of NI. In general, screening involves performing clinical diagnosis and laboratory analysis. Any patient currently or previously hospitalized is considered at risk if he or she presents with signs and symptoms of infection. Analyses of stools or clinical specimens (for CDADs), blood, nasal smears, blood cultures, and laboratory tests can be used to detect NIs, in accordance with predefined surveillance protocols for each NI, in symptomatic or even asymptomatic patients (35–39).

**iv) Additional precautions**. Besides the three above-mentioned basic practices, additional precautions must be taken when an NI is declared. While they depend on the infection detected, they involve, among other things, implementing isolation measures and contact precautions for patients who are carriers or infected (40). In the event that a major outbreak of an NI is declared, CBPs must be applied intensively and additional care team meetings and resources added for as long as it continues (41).

### An Operational Framework and Research Questions

Prior to embarking on an EA, it is important to understand that any interpretation of economic studies must consider three elements: the analytical perspective, the time horizon, and the allocation of healthcare resources and interventions, taking into account the prior condition of the patient (12). The *analytical perspective* determines what costs are included in the calculations. The study can be done from the patient, hospital, or societal perspective. For example, from a hospital perspective, medical costs will not include costs related to the patient post-discharge nor those related to lost productivity due to hospitalization. The *time horizon* defines the time frame for measuring medical costs. As well, the *stage and severity of illness*, comorbidities, risk factors, admitting diagnosis, and length of stay all influence the costs of care (12, 42, 43). Conducting an EA of an NIPC program thus involves examining questions about the quality of management and prevention, as well as about the safety of care. Thus, as Finkler (1993, 1996) states, the cost of managing quality takes into account the cost of investing in preventive measures and the costs associated with poor quality or with problems experienced (44, 45). According to Finkler, a certain level of quality could be achieved by investing in prevention. Thus, there is a breakeven point, referred to as “the optimum,” above which prevention could increase with quality. Thus, based on Finkler's model (1993, 1996), an EA of the NIPC program, using CBPs, involves answering the following questions:

- ***What is the cost of investing in prevention through best clinical practices in*** ***NIPC?***
- ***What are the costs of NIs?***
- ***What is the optimal breakeven point for measuring return on investment when*** ***comparing prevention intervention costs against potential benefits?***

### Settings and Project Scope

Because NI incidence varies according to types of facilities and of patients present in hospitals (23), and in keeping with the standards for reporting statistics by region in the documents of Provincial NI Surveillance Group (SPIN) of Quebec (35–38), this project will be conducted in two Quebec hospitals, one nonUniversity and the other University affiliated. As such, one of the selected sites will be a nonUniversity hospital included in the CISSS des Laurentides (integrated health and social services centre for the Laurentians region), and the other, a University-affiliated hospital included in the CIUSSS (integrated University centre for health and social services) of the Saguenay–Lac-Saint-Jean region.

Four reasons prompted the choice of these two regions. First, these regions are *de facto* part of this project, given the involvement of researchers and professionals with NIPC expertise affiliated with universities and health centers in these regions. Second, in the Laurentians region, the incidence rate of *Clostridium difficile-associated diarrhea* (CDAD) has been comparable to the provincial average over the past 5 years (35). The Saguenay–Lac-Saint-Jean region has an incidence rate of CDAD slightly below the provincial average for the same period. Third, the Laurentians region has an incidence rate of nosocomial VRE colonization higher than the provincial average over the past 5 years (with the exception of 2016–2017), while, over the same period, this rate has been significantly lower than the provincial average in the Saguenay–Lac-Saint-Jean region (36). Lastly, the Laurentians region is a CISSS while the Saguenay–Lac-Saint-Jean region is a CIUSSS. These are two different case types that will help cover a fairly broad spectrum of costs in Quebec hospitals. A nonUniversity hospital will be selected from the CISSS, and a University-affiliated hospital will be selected from the CIUSSS. It is important to note that two co-investigators (Sandra Boivin and Catherine Larouche) will facilitate obtaining the consent and participation of the two hospitals, each in their respective regions. Moreover, a similar project has been submitted to the multicenter ethics committee of the Laurentians CISSS with the involvement of the Saguenay–Lac-Saint-Jean CIUSSS.

### Project Components

The proposed project has four components that each provides deliverables:

#### Component 1: Construction and Content Validation of an Observation Grid for Measuring the Costs of CBPs Associated With NIPC

The best clinical practices that will be studied are those described in guidelines for the prevention of CDAD, MRSA, VRE, and *carbapenemase-producing Enterobacteriaceae* (CPE, or CPGNB).

***Methods****:* A “time-motion” observation grid (46, 47) was constructed to measure the time taken (in seconds) by health workers to perform each CBP (hand hygiene, hygiene and sanitation, screening, and additional precautions). This grid was developed in accordance with the strategies and methods for measuring compliance in each of the CBPs recommended by the Public Health Institute (INSPQ) of Quebec (29, 30). The grid was constructed according to the algorithm developed by the project team, which is presented in Appendix 2. Using a Delphi approach, the grid will be validated by 18 experts (six field experts from CISSS des Laurentides, six field experts from CIUSSS du Saguenay–Lac-St-Jean, and four content experts). The field experts are NIPC professionals, microbiology and infectious disease specialists, as well as professionals in hygiene and sanitation. The content experts have been selected for their expertise in the field of NIPC. The degree of content validity of each section of the grid will be measured by means of the content validity index (CVI) by retrieving the percentage of statements rated 3 and 4 (number of statements rated 3 and 4/total number of statements in the grid) (40–50). A CVI ≥0.80 will show that the grid is acceptable and its content valid with minor corrections (49). If it is <0.80 in any section of the grid, that section will undergo major revision and then be resubmitted to the same experts for assessment. Referring to their previous comments and suggestions, they will be asked to reassess it, based on the previous ordinal scale, until a CVI ≥0.80 is obtained for each section of the observation grid. At least two rounds will be required.

***Deliverable 1***: **Production and validation of a standardized grid**, which can be used in all general and specialized (medical and surgical) physical health services units in Quebec to measure the costs of the four practices: hand hygiene, hygiene and sanitation, NI screening, and additional precautions.

#### Component 2: Use of the Constructed and Validated Grid to Assess the Costs of the Four CBPs in a CISSS Hospital in the Laurentians Region and a University-Affiliated CIUSSS Hospital in the Saguenay–Lac-Saint-Jean Region

In each of the two **hospitals**, two units from the general and specialized (medical and surgical) physical health services will be randomly selected for the study. A prospective investigation (48) will be conducted in the four study units to collect information on CBPs through direct observation.

***Methods:*** In line with CBP compliance assessment methods (29, 30), a sample of practitioners, a sample of patient records, and a sample of patients will be selected for each type of CBP studied over the course of the year (April 1, 2019, to March 31, 2020).

For *hand hygiene*, in each of the four units of the two selected hospitals, three physicians, three nurses, three nursing assistants, and three patient attendants will be randomly selected from the day and evening shifts. During this period, for each hand hygiene opportunity, time taken (in seconds) and products used will be systematically recorded. For *hygiene and sanitation*, in addition to the above-mentioned practitioners, three hygiene and sanitation workers will also be randomly selected and observed during disinfection (daily and terminal) of the ordinary rooms and/or for additional precautions.

Each of the 15 workers will be shadowed by a research nurse observer duly trained for this purpose, for 3 h in their shift. Given the heavy volume and activity during the day, two out of three workers will be randomly selected during the day shift (10 during the day and five in the evening).

As recommended by INSPQ (29, 30) for the assessment of CBP compliance, observations will be made during six 2-week observation periods conducted every 2 months over the course of 1 year.

A total of **15** **×** **6** **=** **90 workers** (3 × 6 = 18 physicians, 18 nurses, 18 nursing assistants, 18 patient attendants, and 18 hygiene and sanitation workers) will be observed per unit during 1 year. Thus, for two units in one hospital, 180 workers will be observed during the year, i.e., **360 workers** for the two hospitals. It is possible, due to random selection, that a worker may be observed more than one time during the year.

*Practices related to additional precautions* will be systematically observed when applied by the workers.

With regard to *screening*, as suggested by the INSPQ (29, 30), in each 2-week observation period, to be conducted six times over the year, 15 clinical records of patients screened (at admission or during hospitalization) for MRSA, VRE, and CPGNB (i.e., five records for each of the three screened NIs) will be randomly selected from the total number of patient records screened in the two units of each hospital. In addition, five records with diagnostic tests for CDAD detection will also be randomly selected from the records of patients with diarrhea in the two units of each hospital. Thus, 20 files × 6 observation periods, or 120 patient files studied per hospital, will be reviewed during the year, i.e., 240 patient files for both hospitals in total. All screening tests and diagnostic tests (related to CDAD) performed on a selected patient will be noted. The screening and diagnostic testing algorithms for each NI (51–54) in Appendix 3 will be used as models. By associating a cost with each test, the total cost of patient screening will be calculated. An average annual cost will be estimated.

With regard to *training and information sessions and any meetings that may be organized* by the NIPC service of the hospital in the units studied, a dashboard will be maintained throughout the observation year to systematically record participants, their professional profiles, trainers, instructors, duration of meetings, and equipment used. Finally, one representative (e.g., from the central supplies service) from each hospital (two in total) will provide information on unit prices and depreciation for each of the products and materials used. Thus, in addition to the activities of NIPC nurses (the grid), awareness-raising and information campaigns, and training activities for health personnel in the unit, information will be collected at weekly, monthly, and annual meetings; management and coordination activities related to NIPC in the care units; evaluation activities; and investments made (as needed).

***Deliverable 2*: An estimate of the average annual cost of each CBP** (hand hygiene, hygiene and sanitation, screening, additional precautions) and the average annual overall cost of actions implemented for NICP (including training and information activities), whether during an outbreak period or not, in a teaching hospital and a non-teaching hospital. To this end, costs will be estimated on a 1-year horizon in Canadian dollars (CAD) and from a healthcare facility perspective (costs related only to hospital activities). The following cost indicators will be estimated (in days) per unit: the cost of an observation; the cost of a CBP; the cost per worker; and the overall cost of the four CBPs considered together in one unit. *The cost of an observation will be calculated as follows: (Time spent [in minutes] on an observation* × *the hourly wage of the worker [in minutes)* × *24 h* + *(Depreciated cost of the product or material used (in days) during the observation). The cost of a CBP (in days)* will be the sum of the costs of the observations made. The overall cost will be the sum of CBP costs and costs related to meetings, training sessions, and awareness-raising campaigns. Sensitivity analyses will be carried out by varying the hourly wages of workers from minimum to maximum, according to their salary scales (lowest and highest salary scales). For physicians, the rate for a regular consultation for a patient at risk of infection will be applied.

#### Component 3: Assessment of the Economic Burden Attributable to the Occurrence of Nosocomial Infections in Two Quebec Hospitals

While the costs of NIs in healthcare facilities have been sufficiently analyzed in Western scientific literature (12, 55), to our knowledge, there are currently no empirical studies in Quebec on the financial costs associated with the four NIs most closely monitored in the province (CDAD, MRSA, VRE, and CPGNB). Complementing the epidemiological surveillance carried out by SPIN, the results of Component 3 will provide decision-makers in the Quebec healthcare network with information on the health-related and economic benefits of strengthening the NIPC program.

**Methods:** Assessing the economic burden of NIs involves estimating, from the healthcare facilities perspective, the additional financial cost of each of the four Nis during a care episode. As such, it includes the additional financial costs attributable to the increase in overall care consumption, the extension of the length of stay, the consumption of drugs, and the increase in laboratory tests caused by the occurrence of an NI (56). This component is based on a matched case-control design of patients with an NI (colonized or infected patients: cases) with similar patients who have not contracted an NI (controls) for a given period of time. The advantage of using such a design is that it is suitable for infrequent diseases such as NIs, and it also allows comparisons to be made between similar groups that are differentiated only by the presence of an illness (57).

*Population*: Included: Patients 18 years of age and over hospitalized for at least 72 h in the four units between April 1, 2019, and March 31, 2020. A patient readmitted either for a new hospital stay or for a complication related to the initial admission will be considered a new patient. Patients will be followed for a maximum of 30 days on the unit. A *case* is a patient who has contracted one of the four NIs studied. A *control* is a similar patient who did not develop an NI during the same care episode. *Matching*: Propensity score matching will be used, followed by 1:1 nearest neighbor matching (58–60). As in D'Amour et al. (9) and Tchouaket et al. (56) the cases and controls will be matched according to age, sex, risk factors, admitting diagnoses, comorbidities, date of admission to the unit, and severity of illness on admission as measured by the Charlson index (61). *Sample size*: All cases of each of the NIs occurring between April 1, 2019, and March 31, 2020, in the care units of the two hospitals will be included in the study. For MRBs (MRSA, VRE, and CPGNB), a distinction will be made between colonized patients (diagnosed on admission) and patients infected in the hospital. *Data source*: Data from hospital archives containing information on the units studied will be used. A grid (see a model in Appendix 4) will be used to extract the following information: health status of a patient on admission; date of NI occurrence; date of discharge of the patient from the unit; care and services consumed during the period in the unit; medical procedures performed by physicians; interventions carried out on the patient (professional involved, date, time spent); drugs consumed; laboratory tests performed; and imaging services used.

***Deliverable 3*: An estimate of the care and services consumption cost for each patient group (cases and controls) over a 1-year horizon and from a health facility perspective**, and **cost comparison between cases and controls to determine the financial burden (additional cost) generated by the occurrence of an NI**. First, the time spent by nonphysician professionals to perform each intervention on a patient will be multiplied by the hourly wage of the professional in question to estimate the *cost per nonphysician professional intervention per patient*. Data on medical procedures performed for the benefit of a patient (according to the profile of the physician) will be collected, and, based on RAMQ and AP-DRG data, the *medical (physician) costs per patient* associated with each procedure will be estimated. Laboratory and medical imaging fees will be determined from the MSSS price list to estimate the *cost of diagnostic tests* per patient. Lastly, the prices of each drug consumed (based on actual drug costs at the pharmacy, without insurance deduction) will be used to estimate the *cost of drugs consumed per patient*. *The cost of care and services consumed per patient will be the sum of the cost of nonphysician professional interventions received, the cost of medical procedures, the cost of diagnostic tests, and the cost of drugs consumed per patient*. Second, the additional cost generated by the occurrence of an NI will be determined by estimating the average additional cost generated by the presence of an NI (additional cost) after matching propensity scores *via* the *average treatment effects on the treated (ATT)* parameter. STATA 14 statistical software will be used for this purpose, and the 95% confidence interval for the ATT will be determined, using the bootstrap method by determining the 2.5th percentile and 97.5th percentile of the ATT series obtained after resampling and matching in 1,000 iterations (56, 62, 63). Sensitivity analyses will be conducted in both steps by varying the annual hourly wage of nonphysician professionals from minimum to maximum according to their salary scales.

#### Component 4: Determination of the Optimal Breakeven Point for NI Prevention Practices

Associated with objective three of the project, this more analytical component is aimed at measuring the return on investment in NIPC by comparing the costs of implementing CBPs against the potential benefits. This will involve estimating the potential benefits associated with the reduction of NIs *via* CBPs and determining the optimal breakeven point at which implementing CBP activities associated with NICP becomes profitable.

**Calculation of potential benefits:** If the use of CBPs associated with NIPC leads to an X% reduction in NIs and, consequently, a reduction in NI costs related to management of carrier or infected patients, economic benefits will be observed (64). Taking into account an X% reduction in NI prevalence due to an NIPC program, the benefit (B) is calculated as follows: B = (1 – X%) × total cost of NIs. Because the results in the literature are divergent, with the proven effectiveness of NIPC programs ranging from 0 to 50% (31), we will conduct a sensitivity analysis to estimate benefits by varying X%. Determination of the breakeven point: The cost-effectiveness of the NIPC program in relation to CBPs will be assessed by comparing the benefits of the NIPC intervention with the costs of implementing CBPs. The benefit–cost ratio (B/C) will be calculated. This will provide information on the savings achieved, in 2019 dollars, for each dollar invested in the NIPC program related to implementing CBPs. The program will be considered cost-effective if the B/C ratio is >1. This ratio will be calculated by varying the percentage X of NI reduction from 0 to 50%. Sensitivity analyses will also be conducted to determine the breakeven point for the NIPC program in relation to the four CBPs.

## Discussion

### Knowledge Transfer and Results Use

Before presenting the knowledge transfer strategies, a brief overview of the different stakeholders involved in this proposal is in order. The *Université du Québec en Outaouais* is represented by the principal investigator (Prof. Eric Tchouaket), a researcher in health economics and holder of an FRQS junior 1 career award (2017–2021); one co-investigator, a physician and public health specialist (Dr. Drissa Sia); and one co-investigator, a nursing research fellow and former NIPC consultant (Dr. Sylvain Brousseau). The *Université de Montréal* is represented by one co-investigator, a nurse specialized in time–motion grid development and validation (Dr. Kelley Kilpatrick). The *CISSS des Laurentides* is represented by one co-investigator, the overall coordinator of the project, a clinical nurse specialized in NIPC (Ms. Sandra Boivin). The *CIUSSS du Saguenay–Lac-Saint-Jean* is represented by one co-investigator, an NIPC senior consultant (Ms. Catherine Larouche). The *Montreal Heart Institute* is represented by one co-investigator, a coordinator in hygiene and sanitation (Mr. Bruno Dubreuil). The *INSPQ* is represented by one co-investigator, a consultant in nursing care, immunization, and nosocomial infections from the Department of Biological Risk and Occupational Health (DRBST) and NIPC expert (Ms. Natasha Parisien). The project has been endorsed by the NICP leaders in both regions of the study. Added to this team are the *Réseau de recherche en interventions en sciences infirmières du Québec (RRISIQ)*, which partially funded component 1; and the *Fonds de Recherche Québec en Santé (FRQS)*, which provided a career award to the principal investigator from 2017 to 2021 to conduct components 1, 2, and 3 of this proposal. The *MSSS*, which is the organization that will implement and conduct the work related to target 22 of its action plan, is theoretically an *ex officio* stakeholder in this proposal. The team also includes research assistants (one statistician and one computer scientist), research nurses (responsible for data collection), and students (master's or doctoral).

Knowledge transfer is central to this project proposal. The aim is to present clearly how the results will be used and transferred to the various stakeholders and key actors in the Quebec health network (policy-makers, including the MSSS; the INSPQ *via* the SPIN and the CINQ (Comité des infections nosocomiales du Québec); the CEOs of the CISSS and CIUSSS and their boards of directors; nursing directors; health professionals; and researchers).

First, the partnership built through this proposal will foster ongoing interactions among decision-makers, researchers, managers, and practitioners. It is based on a deliberative approach that incorporates knowledge translation as an integral part of the research process. The knowledge generated will be discussed, adapted, or negotiated within the teams to be applied in specific contexts. Regular meetings will be held with all stakeholders to organize data collection, mobilize the staff of the institutions involved, finalize the methodology, and discuss the results. Collaborations with the public health and nursing departments of the CISSS des Laurentides and the CIUSSS du Saguenay–Lac-Saint-Jean, as well as with the experts participating in the study (component 1, the Delphi process) will provide opportunities to design and implement knowledge transfer activities.

Second, the time–motion observation grid developed here, whose content is validated in component 1, can be used by the MSSS for the systematic collection of information related to CBP costs. To this end, a web and mobile application, the *AnéPCI*, will be developed by the computer technician that will allow the grid to be viewed on computers, tablets, and telephones since the collection of observation data (component 2) will be done *via* tablets. The platform will be free of charge, but only the principal investigator will grant access to those wishing to consult it. At the same time, the stakeholders in this project (MSSS, INSPQ, researchers, CISSS des Laurentides, and CIUSSS du Saguenay–Lac-Saint-Jean) will have direct access to the platform (secure access). The platform could be used for technological transfer of results and flexible use of the grid in the case of an eventual Quebec-wide scale up to the other CISSSs and CIUSSSs of the network.

Third, the results of component 2 will quantify the costs of implementing CBPs related to NIPC. Since component 2 will be carried out during the same period as component 3 (April 1, 2018, to March 31, 2020) in the same units of the two hospitals studied, the costs of implementing NIPC (component 2 deliverable) will be compared with the costs of managing patients who are carriers or infected by an NI (deliverable 3). This comparison will provide an opportunity to discuss the cost-effectiveness of the NIPC program through the implementation of CBPs.

Once completed, this project will also provide information on the true cost of CBPs in NIPC, i.e., the costs of both basic practices and additional precautions (component 2). In addition, it will help to determine the cost of a patient colonized or infected with CDAD, MRSA, VRE, or CPGNB. *For decision-makers*, knowledge of infection control costs will allow them to make decisions based on data that represent what is currently being done in facilities within the Quebec health and social services network. This project will allow the MSSS to estimate the cost of its orientations more accurately and to be able to invest human, material, and financial resources in NIPC in the right places and at the right time, using financial resources made cost-effective by the NIPC program. It will, thus, be able to invest in a timely manner in the nurse/patient ratio in additional precautions; nurse ratios for infection prevention; the training of NIPC nurse consultants or specialists; equipment purchases for patient care or for hygiene and sanitation; information and awareness-raising campaigns, etc. *CEOs* and boards of directors in CISSSs and CIUSSSs will be able to make decisions on investments to avoid repeated outbreaks (e.g., adding dedicated equipment in rooms; adding dedicated staff to a cohort; maintaining an empty bed in a cohort of patients who are carriers or infected with MRBs for 24–48 h to avoid bed transfers and admit them promptly, etc.). They will know the financial implications of not having NIPC measures in their budgets.

Knowledge of NIPC cost-effectiveness data should encourage *nursing directors (DSIs)* to maximize NI prevention measures to reduce cases of infection. *Professional practitioners* will be informed about the financial consequences of not applying CBPs in hand hygiene, hygiene and sanitation, and additional precautions. This will provide them with a better understanding of the situation so they can work more closely with managers to ensure the quality and safety of patient care.

Fourth, throughout the study, results of each component will be made available to targeted audiences through a variety of channels: research reports on each component; scientific and review articles; and oral and poster presentations. Appropriate forms of dissemination will be defined in collaboration with managers and decision-makers. All of the stakeholders of the study will be encouraged to make presentations in their communities and to share their opinions on issues related to applying the results of each component of the study.

Fifth, this project will foster the development of more economic analyses in public health and nursing related to infection prevention and control. It will contribute to the training of master's or doctoral students who will be recruited to further develop components 2 and 3. Two research nurses will also be recruited and trained to administer the observation grid, using the platform constructed.

## Caution in Interpreting Results

Economic analysis is an approach that provides information on the financial benefits of investing in an intervention. It complements the epidemiological surveillance already underway. Decisions about implementing an intervention cannot depend solely on the results of this type of analysis. Other sociosanitary, ethical, political, and even environmental dimensions also need to be taken into account.

## Time Frame

This proposal covers the period from January 1, 2018, to December 31, 2020.

## Consent for Publication

Only sociodemographic data will be collected from the participants. Any image and clinical data will be collected. The observation will be done by eye contact, and the time spent for each activity will be counted, using a stopwatch developed in a web/mobile application.

## Ethics Statement

This research protocol has been approved (see supplementary file) by the Multicentre Research Ethics Committee of Centre intégré de santé et de services sociaux de l'Outaouais (CISSSO) and accepted by Research Ethics Committee of the Université du Québec en Outaouais. All the participants will provide informed written consent. They will sign the consent form before the beginning of the observation.

## Author Contributions

ET, SBo, CL, NP, BD, SBr, KK, DS, C-AD, AB, and RB made substantial contributions to study conception and design for this research protocol. All the authors were involved in drafting and making revisions for critical intellectual content in the manuscript. All the authors gave final approval of the version to be published.

## Conflict of Interest

The authors declare that the research was conducted in the absence of any commercial or financial relationships that could be construed as a potential conflict of interest.

## Abbreviations

AB: Astrid Brousselle
ATT: Average treatment effects on the treated
BD: Bruno Dubreuil
CAD: Carl-Ardy Dubois
CBP: Clinical best practice
CDAD: Clostridium difficile-associated diarrhea
CISSS: Integrated health and social services center for the Laurentians region
CIUSSS: Integrated University center for health and social services
CL: Catherine Larouche
CPE, or CPGNB: Carbapenemase-producing Enterobacteriaceae
CVI: Content validity index
DS: Drissa Sia
ET: Eric Tchouaket
HAS: Hydroalcoholic solutions
INSPQ: Quebec's Public Health Institute
KK: Kelley Kilpatrick
MRSA: Methicillin-resistant staphylococcus aureus
MSSS: Quebec's Ministry of Health and Social Services
NI: Nosocomial infection
NIPC: NI program for the prevention and control
NP: Natasha Parisien
OECD: Organisation for Economic Co-operation and Development
ROX: Roxanne Borgès Da Silva
SB: Sandra Boivin
SBR: Sylvain Brousseau
SPIN: Quebec's Provincial NI Surveillance Group
VRE: Vancomycin-resistant enterococcus
WHO: World Health Organization.
