# Supplementary material for: Economic Analysis of the Prevention and Control of Nosocomial Infections: Research Protocol
Source: Front Public Health. 2021 Jul 7;9:531624. doi: 10.3389/fpubh.2021.531624 (PMC8292614; doi:10.3389/fpubh.2021.531624)
Supplement: Supplementary file 1 [file Table_1.DOCX]

**Appendix 1**. Project proposal framework based on best clinical practices.

- Education, awareness, training and motivation regarding hand hygiene

- Administrators’ involvement and support for strategies related to hand hygiene

- Surveillance regarding hand hygiene

- Cleaning and frequent disinfection of at-risk areas

- Protection of floors and surfaces

- Training and information about hygiene and sanitation

- Protocol and intervention techniques

- Placement and accommodation (isolation)

- Use of personal protection equipment

- Management of outbreak

Compliance with clinical protocols according to type of infection: CDAD, MRSA, VRE, CPGNB

**Reduction of nosocomial infections: CDAD, MRSA, VRE, CPGNB**

**NIPC
Program**

**Best clinical practices for NIPC**

Hand hygiene

Hygiene and sanitation of surfaces and equipment

Screening on admission or during hospitalization of symptomatic patients (carriers or infected)

Basic and additional precautions
