# Supplementary material for: Economic Analysis of the Prevention and Control of Nosocomial Infections: Research Protocol
Source: Front Public Health. 2021 Jul 7;9:531624. doi: 10.3389/fpubh.2021.531624 (PMC8292614; doi:10.3389/fpubh.2021.531624)
Supplement: Supplementary file 3 [file Table_3.DOCX]

## **Appendix 3.** Screening algorithms for the four NIs (CDAD, MRSA, VRE, CPGNB)

**Screening algorithm for CDAD diagnostic testing**

Symptomatic patient

Sampling

Analysis in the
hospital laboratory

Positive

Negative

Additional precautions for contact

Continue follow-up according to the health facility protocol

**Symptomatic user:** Presence of diarrhea (at least 3 watery or semi-liquid stools that take the form of a container within 24 hours **AND** diarrhea lasting more than 24 hours with no other obvious cause) or toxic megacolon **AND** laboratory confirmation of the presence of a toxin or a *C. difficile* toxin gene (A or B).

**OR**

In patients who have chronically soft or watery stools, change in stool characteristics (increase in frequency or quantity, or change in consistency) with no other obvious cause other than what is usual for the patient (e.g. Crohn's disease) **AND** laboratory confirmation of the presence of a toxin or a *C.* *difficile* toxin gene (A or B).

**Sampling:** According to the technique used in the study facility.

**Additional precautions:** As described in the guidelines on prevention and control of nosocomial diarrhea associated with *Clostridium difficile* in Québec (INSPQ, 2005).

**Source:** Institut national de santé publique du Québec. *Prévention et contrôle de la diarrhée nosocomiale associée au Clostridium difficile au Québec:* *Lignes directrices pour les établissements de soins* (3rd ed.). Montreal: INSPQ; 2005.

**Screening algorithm for MRSA**

Admission: patient
meets criteria

Sampling

Analysis in the
hospital laboratory

Positive, or
known MRSA

Negative

If hospitalized ≥ 1 week, repeat sampling weekly during the hospital stay

Additional precautions for contact

Sampling

Positive

Negative

**Screening criteria on admission:**

- Direct transfer from one hospital to another (stay >24 hours)
- Arrival from a CHSLD (long-term care facility)
- Arrival from a rehabilitation unit or centre
- Known carrier of MRSA
- History of previous hospitalization in the past 3 years including in the hospital where the patient is currently admitted
- Other clienteles depending on local epidemiology

**Sampling:** According to the technique used in the study facility.

**Additional precautions for contact:** As described in the guidelines on prevention and control of methicillin-resistant *Staphylococcus aureus* (MRSA) infections in Québec (INSPQ, 2006).

Additional precautions for contact

**Source:** Institut national de santé publique du Québec*. Mesures de prévention et de contrôle des infections à Staphylococcus aureus résistant à la méthicilline (SARM) au Québec.* (2nd ed., interim version). Montreal: INSPQ; 2006.

**Screening algorithm for VRE**

Admission: patient
meets criteria

Sampling

Laboratory analysis in the hospital

Positive, or
known VRE

Negative

If hospitalized ≥ 1 week, repeat sampling weekly during the hospital stay

Additional precautions for contact

**Screening criteria on admission:**

- Know VRE carrier
- History of hospital stay ≥ 24 consecutive hours in the previous 12 months in any care setting
- Direct transfer from one hospital to another (stay >24 hours)

**Sampling:** According to the technique used in the study facility.

**Additional precautions for contact:** As described in the guidelines on prevention and control of vancomycin-resistant *enterococci* in acute-care settings in Québec (INSPQ, 2012).

Sampling

Positive

Negative

Additional precautions for contact

**Source:** Institut national de santé publique du Québec. *Mesures de prévention et de contrôle de l’entérocoque résistant à la vancomycine dans les milieux de soins aigus : avis et recommandations*. Montreal: (INSPQ); 2012.

**Screening algorithm for CPGNB**

Admission: patient meets criteria

Day 14

Day 7

Day 0

Sampling

Sampling

Sampling

Laboratory analysis in the hospital

Negative

Suspect

Laboratory analysis in the hospital

Additional precautions for contact

Negative

Suspect

Laboratory analysis in the hospital

Additional precautions for contact

Negative

Suspect

Additional precautions for contact

Hospital laboratory sends the specimen to the LSPQ (Québec public health laboratory)

Negative

Positive

Stop additional precautions for contact

Additional precautions for contact

**Screening criteria on admission:**

- Patient transferred directly from a health facility outside Québec
- Patient who was hospitalized or received care in a health facility outside Quebec within the past year.
- Patient transferred directly from a health facility in Quebec (hospital, CHSLD, or rehabilitation centre) with active outbreak
- Patient who, within the past 3 months, was hospitalized or spent ≥ 24 hours in a Quebec health facility (hospital, CHSLD, or rehabilitation centre) on the list of facilities having experienced an EPC outbreak (MRB Advisory list)

**Sampling:** According to the technique used in the study facility and LSPQ analysis protocols

**Additional precautions for contact:** As described in the knowledge transfer activity (INSPQ webinar, November 8, 2017) entitled *Entérobactéries productrices de carbapénémases : mesures de prévention et de contrôle pour les soins aigus*.

**Source:** Institut national de santé publique du Québec. *Entérobactéries productrices de carbapénémases : mesures de prévention et de contrôle pour les soins aigus*. INSPQ knowledge transfer activity (webinar) presented by Dr. Josée Massicotte, November 8, 2017.
