# Supplementary material for: Economic Analysis of the Prevention and Control of Nosocomial Infections: Research Protocol
Source: Front Public Health. 2021 Jul 7;9:531624. doi: 10.3389/fpubh.2021.531624 (PMC8292614; doi:10.3389/fpubh.2021.531624)
Supplement: Supplementary file 4 [file Table_4.DOCX]

## **Appendix 4.** Data extraction grid used to extract surveillance data on hospitals’ NIPC services

**I. DATA RELATED TO THE PATIENT’S CHART**

Facility code __ __ Unit code __ __ Patient chart # __ __ __ __ __

Date of analysis __ (dd) __ (mm) ____ (yyyy)

Patient code __ __ __ __ __ __

Code of the data extraction agent ________

**II. CONFIRMATION OF CRITERIA**

**Inclusion criteria**

Check (X) all that apply

❏ ≥ 18 years

❏ ≥ 72 hours stay on the target unit

❏ Patient present on the unit during the period targeted for the study (up to a maximum of 30 days)

**If not met:**

❏ File rejected (chart review stopped)

Reason: _________________________________________________________________

**III. DATA RELATED TO PATIENT ON ADMISSION**

Age of patient on admission to the unit __ __ years

Patient sex ❏ M ❏ F

Date of patient’s admission to the unit __ (dd) __ (mm) ____ (yyyy)

Date of patient’s admission to the facility (if different from the above date) __(dd)__(mm)____(yyyy)

Date of patient’s discharge from the unit __ (dd) __ (mm) ____ (yyyy)

**IV. DATA RELATED TO PATIENT’S HEALTH STATUS (SEVERITY) ON ADMISSION**

***Comorbidities*** *(adapted from Charlson et al.). Check (X) all responses that apply:*

❏ Myocardial infarction ❏ Heart failure ❏ Peripheral vascular failure

❏ Cerebrovascular disease ❏ Dementia ❏ Chronic lung disease

❏ Connective tissue disease ❏ Ulcerative disease ❏ Hepatopathy

❏ Diabetes ❏ Hemiplegia ❏ Moderate to severe renal disease

❏ Diabetes with organic lesions ❏ Neoplasms of all origins ❏ Moderate to severe hepatopathy

❏ Metastatic solid tumors ❏ AIDS

**Other comorbidities.** Check (X) all responses that apply:

❏ Obesity ❏ Alcoholism ❏ Drug use ❏ Depression ❏ History of smoking

❏ Illiteracy ❏ Cognitive disorder (Alzheimer’s, schizophrenia…) ❏ Language spoken by patient different from that of staff

**Other factors to be considered**

Did the patient present, on admission, one or more of the following infections targeted by the study (patient carrier or colonized)?

❏ No

❏ Yes -- Check (X) all that apply

❏ CDAD ❏ MRSA ❏ VRE ❏ CPGNB

**Admitting diagnosis:_____________________________________________________________**

**Primary diagnosis:_________________________________________________________________**

**Secondary diagnoses:____________________________________________________________**

**VI. DETECTION OF NOSOCOMIAL INFECTIONS (ATTENTION: use one sheet for each NI)**

**Patient code __ __ __ __ __ __**

**Type of nosocomial infection (NI) (Check the box that applies) :**

❏ CDAD ❏ MRSA ❏ VRE ❏ CPGNB

**Date of occurrence of infectious episode ___ (dd) ___ (mm) ____ (dddd)**

**V- DATA ON CARE AND SERVICES CONSUMPTION TO BE EXTRACTED FOR COST CALCULATION**

Fill in the following embedded table (consisting of four tables) in detail (in the analysis, the focus will be on the 30 days of hospitalization for each patient)

| **1. Lists of all non-medical interventions received in 30 days** | Date done or administered (DD/MM/YYYY) | Duration of intervention  (in seconds) | Title of the non-physician practitioner who performed the intervention |
| --- | --- | --- | --- |
| Intervention i (i = 1,2,3,…)* |  |  |  |
| **2. Lists of drugs prescribed or consumed in 30 days** | Date of prescription or consumption  (DD/MM/YYYY) | Unit price in the pharmacy  (in CAD dollars) |  |
| Medication i (i = 1,2,3,…) |  |  |  |
| **3. Lists of tests prescribed or performed in 30 days** | Date of prescription or performance  (DD/MM/YYYY) | Price of test in the laboratory  (in CAD dollars) |  |
| Exam i (i = 1,2,3,…) |  |  |  |
| **4. Lists of medical acts for 30 days** | Date of medical act  (DD/MM/YYYY) | Duration of act  (in seconds) | Cost of act per RAMQ  (in seconds) |
| Medical act i (i = 1,2,3,…) |  |  |  |

*if the intervention, drug, test, or medical act is the same but on different dates, fill in another line.
